# Enrichment of *Streptococcus oralis* in respiratory microbiome enhance innate immunity and protects against influenza infection

**DOI:** 10.1038/s41392-025-02365-x

**Published:** 2025-08-27

**Authors:** Xiaohui Zou, Hongyun Cao, Lizhe Hong, Lijun Suo, Chun Wang, Kang Chang, Yawen Ni, Bo Liu, Bin Cao

**Affiliations:** 1 National Center for Respiratory Medicine; State Key Laboratory of Respiratory Health and Multimorbidity; National Clinical Research Center for Respiratory Diseases; Institute of Respiratory Medicine, Chinese Academy of Medical Sciences; Beijing Key Laboratory of Surveillance, Early Warning and Pathogen Research on Emerging Infectious Diseases; Laboratory of Clinical Microbiology and Infectious Diseases, Department of Pulmonary and Critical Care Medicine, Center of Respiratory Medicine, China-Japan Friendship Hospital, Beijing, China; 2 Department of Clinical Microbiology, Pulmonary and Critical Care Medicine, Zibo City Key Laboratory of Respiratory Infection and Clinical Microbiology, Zibo City Engineering Technology Research Center of Etiology Molecular Diagnosis, Zibo Municipal Hospital, Zibo, China; 3 Chinese Academy of Medical Sciences and Peking Union Medical College, Beijing, China; 4 Weifang People’s Hospital, Shandong Second Medical University, Weifang, Shandong Province China; 5 Department of Pulmonary and Critical Care Medicine, Shandong Institute of Respiratory Diseases, The First Affiliated Hospital of Shandong First Medical University, Shandong Provincial Qianfoshan Hospital, Shandong University, Jinan, China; 6 New Cornerstone Science Laboratory, Beijing, China

**Keywords:** Respiratory tract diseases, Infection

## Abstract

Respiratory microbial dysbiosis has been implicated in the occurrence and progression of community-acquired pneumonia (CAP). However, the dynamic variation in the respiratory microbiota and its interaction with the host response remain poorly understood. Here, we performed metagenomic analysis of respiratory and gut microbiota, along with blood transcriptomics, using longitudinally collected samples from 38 CAP patients. CAP patients presented disrupted sputum microbiota at the early, middle, and late stages of hospitalization. Microbial pathways involved in peptidoglycan biosynthesis and immune evasion, particularly contributed by the *Streptococcus* genus, were enriched in CAP patients. Additionally, several *Streptococcus* strains demonstrated correlation between respiratory and gut microbiota in CAP patients. By incorporating host response data, we revealed that *Streptococcus oralis* (SOR) was associated with host pathways involved in the innate immune response to infection, and this microbe‒host interaction was reproduced in a newly enrolled CAP cohort consisting of 22 patients with influenza infection. The host-SOR interaction was validated in a mouse model, where SOR demonstrated protective efficacy against influenza virus infection comparable to that of the well-established respiratory probiotic *Lactobacillus rhamnosus GG*. Preaspiration of SOR in mice significantly mitigated body weight loss, reduced lung inflammation, and lowered viral loads following influenza virus challenge. Host response profiling indicated that SOR priming activated a greater innate immune response at the early stage of infection and that this response resolved timely as the host began to recover. These findings suggest that respiratory commensals play an immune-protective role by inducing a timely innate immune response to prevent CAP progression.

## Introduction

Community-acquired pneumonia (CAP) is a significant public health concern worldwide because of its high morbidity and mortality rates.^1^ CAP is an infection of the lungs acquired outside of hospitals or healthcare settings that affects individuals of all ages, but it is particularly severe in elders, infants, and those with preexisting health conditions.^2^ According to the Global Burden of Disease Study 2021, lower respiratory tract infections cause approximately 2.5 million deaths worldwide in adults, ranking as the fifth leading cause of death globally (fourth when COVID-19-related mortality is excluded).^3^ These findings underscore the urgent need to develop effective strategies for the prevention and treatment of CAP.

The respiratory microbiome plays a critical role in the health and disease of the respiratory tract.^4,5^ As an open system exposed to the external environment, the respiratory tract is inhabited by a diverse array of microorganisms. The microbial composition and biomass vary significantly between the upper respiratory tract (URT) and the lower respiratory tract (LRT), characterized by microbial diversity, complexity, and quantity of the biomass decrease from the URT to the lungs.^4^ Common genera found in the LRT included *Prevotella, Streptococcus, and Veillonella*, which maintains dynamic equilibrium between URT microbial immigration and microbial clearance. Disruption of the balance of these microbial communities, known as dysbiosis, is associated with the development and progression of respiratory diseases, including CAP.^6,7^ Traditionally, respiratory pathogen invasion and expansion are considered the cause of CAP, and eliminating these “causative agents” is the routine therapeutic strategy. This view is grounded in a long-standing dichotomy that classifies microbes as either harmless commensals or harmful pathogens. However, recent advances have led to a shift in this view, suggesting that the lung microbial community may contribute to pneumonia pathogenesis and progression through complex host–microbe interactions. This evolving view supports the investigation of the broader respiratory microbiome—including organisms not classically defined as pathogens—in understanding CAP pathogenesis and host immune modulation.^8^

Various studies have shown that patients with CAP often exhibit reduced microbial diversity, increased bacterial load, and overgrowth of potential pathogenic bacteria.^9^ Several investigations into CAP patients via 16S ribosomal RNA (rRNA) gene sequencing of sputum samples have indicated that *Veillonella* and *Streptococcus* are the most abundant taxa.^9,10^ In patients with sepsis and acute respiratory distress syndrome (ARDS), the lung microbiome is enriched with gut-associated bacteria and is significantly correlated with alveolar TNF-α,^11^ a key mediator of alveolar inflammation in ARDS. For patients with COVID-19 requiring mechanical ventilation, poor clinical outcomes are associated with lower airway enrichment of oral commensals.^7,12,13^ These cross-sectional studies provided detailed alterations in the lower respiratory microbiome in the acute phase compared with that in healthy controls. However, the dynamics of how the respiratory microbiome changed throughout the entire hospitalization period until the recovery stage are still unknown. The five bacteria found in most healthy individuals, which are composed of the genera *Prevotella*, *Streptococcus*, *Veillonella*, *Fusobacterium* and *Haemophilus*, have been proposed as core pulmonary microbiota essential for lung homeostasis.^6^ The lungs of healthy adults are thought to be seeded by oral taxa via microaspiration and mucosal translocation.^14^ Several studies have shown that episodic aspiration with oral commensals can decrease susceptibility to *Streptococcus pneumoniae* in mice through innate immune priming.^15,16^ However, some oral commensals are also enriched in the lower respiratory tract when patients develop CAP,^17^ but how these microbes interact with the host response and their role in disease progression remain unknown.

Emerging research supports that lung microbes can shape local immune tone.^4^ For instance, aspiration of oral commensals has been shown to trigger TLR2-dependent neutrophil responses^15^ or MyD88-dependent Th17 responses,^16^ both of which enhance pulmonary defense against *S. pneumoniae*. Notably, this immunological priming effect persists beyond the transient presence of aspirated microbes and has a lasting impact on host innate immune responses. Yet, little is known about how variations in these microbial populations interact with host immunity during acute respiratory virus infections. In this study, we enrolled CAP patients and longitudinally collected sputum, blood and stool samples to investigate the dynamic variation in the respiratory microbiome throughout the entire hospitalization course to four months post infection. Our findings revealed that the respiratory microbiome in CAP patients exhibited persistent dysbiosis during hospitalization, with enrichment of specific taxa. By integrating the host response and respiratory microbiome profile, we uncovered complex interactions between respiratory commensal microbes and the host immune response in CAP patients. Notably, we identified *Streptococcus oralis* (SOR) as a key commensal species with the potential to activate innate immunity in both CAP patients and a later-enrolled cohort with influenza A virus infection. Intranasal introduction of this bacteria into the respiratory microbiome of mice triggered a rapid activation of innate immune response following influenza A virus challenge and improved mice survival outcomes. Our study delineates the intricate relationship between the respiratory microbiota and host immunity in CAP, highlighting the immunomodulatory potential of specific commensal species in shaping disease outcomes.

## Results

### Patient characteristics

We ultimately enrolled 38 CAP patients for longitudinal sputum collection, with a median age of 68 years (IQR, 60–77), and 21 were females (Table 1, supplementary Table 2). The median age of the 33 healthy controls was 60 years (IQR, 52–65), with 25 being female and 14 being current smokers (supplementary Table 2). Both groups consisted primarily of elderly individuals, although the age difference was statistically significant (*p* = 0.003). No significant difference in sex distribution was detected by the chi-square test (*p* = 0.12). Approximately half of the participants (17 out of 38) received antibiotics prior to specimen collection. The clinical presentation of most patients was mild-to-moderate, with 42.1% requiring no breath support and 50% receiving oxygen supplementation via high-flow nasal cannula. Invasive mechanical ventilation was applied in 3 patients, 2 of whom passed away during hospitalization. A total of 80 sputum samples were collected from the three time points in the acute phase of CAP, and 9 samples were collected during follow-up. SARS-CoV-2 was the most frequently detected pathogen, which was positive in 20 (52.5%) patients, and bacteria were detected in half of these patients concurrently (26.3%). Solo bacterial infection was detected in 6 patients (15.8%). Fungi were detected in 6 patients, with most coinfected with SARS-CoV-2 and/or bacteria. The FluA cohort consisted of 22 patients with influenza A infection, including 12 males, with a median age of 67.5 years (IQR, 50.5–74.5), and exhibited clinical features comparable to those of the CAP cohort (supplementary Table 2).

**Table 1:** CAP patient characteristics

	Overall(*n* = 38)
**Characteristics**	
Age (Years), median (IQR)	68 (60,77)
female sex, *n* (%)	17 (44.7)
**Clinical presentation**	
Breath support	
None, *n* (%)	16 (42.1)
HFNC, *n* (%)	19 (50)
IMV, *n* (%)	3 (7.9)
Time point	
T1(D1～D2), *n* (%)	22 (27.5)
T2 (D3～D4), *n* (%)	25 (31.3)
T3 (D5~discharge), *n* (%)	24 (30)
Follow_up, *n* (%)	9 (11.2)
Pathogen	
SARS-CoV-2, *n* (%)	4 (10.5)
Bacteria,*n*(%)	6 (15.8)
Fungi, *n* (%)	1 (2.6)
SARS-CoV-2/Bacteria, *n* (%)	10 (26.3)
SARS-CoV-2/fungi, *n* (%)	2 (5.3)
Bacteria/fungi, *n* (%)	1 (2.6)
SARS-CoV-2/Bacteria/fungi, *n* (%)	2 (5.3)
Outcome	
Recovery, *n* (%)	36 (94.7)
Death, *n*(%)	2 (5.3)

### CAP sputum metagenomes

**Fig. 1:**
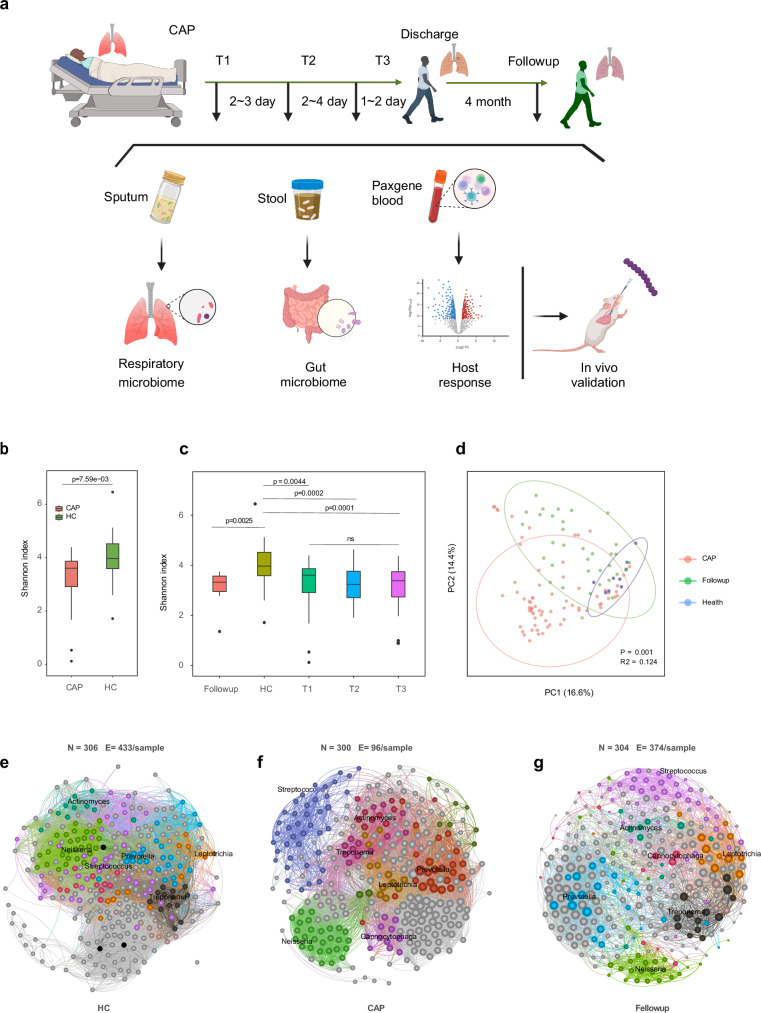
Distinct respiratory microbiome in CAP patients compared with healthy controls. **a** Overview of the study design. Hospitalized CAP patients were enrolled, and longitudinal samples were collected for respiratory/gut microbiome and host response profiling. The host‒microbe interactions detected were validated in a mouse model. **b** Compared with healthy controls, CAP patients expressed lower microbial diversity at admission. **c** The reduced alpha diversity persisted throughout the entire disease course and even 4 months post discharge. **d** Principal coordinate analysis (PCoA) based on the Bray‒Curtis distance revealed significant differences in sputum microbiota composition among CAP patients, healthy controls, and follow-up samples. **e**-**f** Cooccurrence network of microbes in healthy controls (**e**) CAP patients (**f**) and follow-up samples (**g**). Each node represents a microbial species, and edges represent statistically significant co-occurrence relationships. Prominent genera in major modules are colored and annotated on the plot. The numbers of nodes (N) and normalized edges per sample (E/sample) are shown at the top of each panel

Metagenomic sequencing yielded an average of 9.5 × 10^7^ high-quality reads per sample. After host read depletion, an average of 6.0 × 10^6^ clean reads were retained for metagenomic analysis. All the samples yielded a metagenomic coverage of greater than 60%, as estimated by Nonpareil, and were subjected to bacterial taxa profiling. Potential contaminating species in the regent control were excluded from further analysis (supplementary Table 3). We found that CAP patients expressed lower alpha diversity at admission than healthy controls did (Fig. 1b), even after outlier removal (*p* = 0.02). The lower diversity in CAPs persisted throughout the entire disease stage, with no significant difference among the three time points in the acute phase, and was possibly lower than that of healthy controls even at 4 months post discharge (Fig. 1c). The composition of the sputum microbiota differed significantly among CAP patients, healthy controls, and fellow-up samples, as determined via principal coordinate analysis using the Bray‒Curtis distance (Fig. 1d). Moreover, the interactions between bacteria in CAP patients were disrupted and showed a block-like pattern, with less cohesive connections than those in healthy individuals (96 vs. 433 edges per sample). The complexity of these interactions greatly recovered but was still slightly lower than that in healthy controls even 4 months after patient discharge (374 vs 433 edges per sample) (Fig. 1e–g).

### Respiratory microbiota dysbiosis persisted throughout the acute CAP stage

**Fig. 2:**
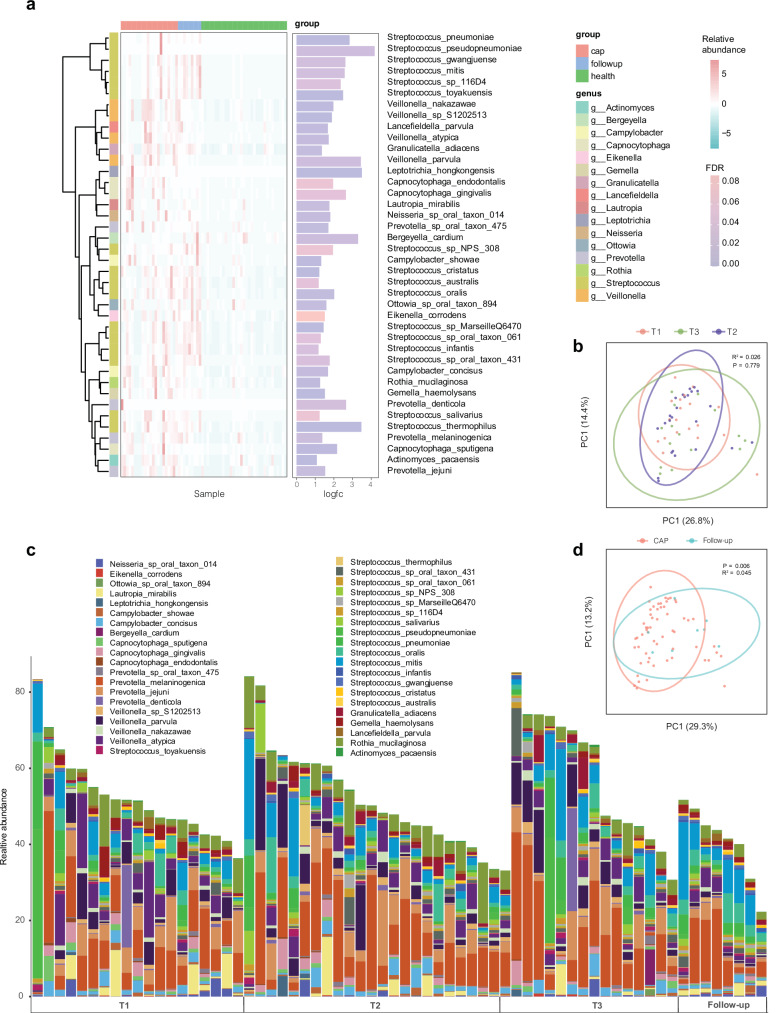
Species-level dynamic variation in the sputum microbiota of CAP patients. **a** The bacterial species significantly enriched in CAP patients compared with healthy controls, grouped by taxonomic classification, with the fold change in abundance shown on the right. **b** PCoA indicated no difference in the microbiota composition at the three time points during the acute CAP stage. **c** The relative abundance of the top 10 bacterial species in each sample at three acute CAP stages (T1, T2, and T3) and at follow-up. **d** PCoA comparing the microbiota composition between the acute CAP phase and the follow-up period

Among the 252 main bacterial species (with relative abundances above 0.01% in at least 20% of the samples) investigated in the cohort, 40 were significantly enriched in CAP patients compared with healthy controls (Fig. 2a). Most of the enriched taxa were commensal microbes frequently observed in the respiratory tract. *Streptococcus* species were the most frequently detected enriched taxa (*n* = 16), followed by *Veillonella* (n = 4) and *Prevotella* (*n* = 4). Notably, these three genera were also among the top four enriched genera in CAP patients in a recent large-scale study involving 917 CAP patients and 25 healthy controls.^10^ The enrichment of *Streptococcus* species in CAP patients persisted into the follow-up period, while the abundances of *Veillonella*, *Capnocytophaga*, and *Rothia* returned to comparable levels to those in healthy controls at the 4-month follow-up.

The microbiota composition at the species level did not differ among the three time points in the acute CAP stage, indicating that the microbiota dysbiosis status was stable throughout the CAP disease course (Fig. 2b). Notably, *Streptococcus* species were the dominant bacteria in most CAP patients, and their high abundance persisted throughout the entire CAP period, even to the follow-up stage (Fig. 2c), with median percentages of 8.9% (IQR 6.1% ~ 13.4%), 9.8% (IQR 6.3% ~ 15.8%), 10.0% (IQR 6.6% ~ 21.4%), and 19.0% (IQR 8.3% ~ 24.9%) in the T1, T2, T3, and follow-up phases, respectively (supplementary Table 1). Another two major taxa highly enriched during the three acute CAP time points, *Veillonella* and *Prevotella*, decreased in abundance as the patients recovered during the follow-up period (supplementary Table 1), resulting in a microbiota composition that differed from that in the acute phase (Fig. 2d).

### Pathogenic microbiota gene modules enriched in CAP patients

Metagenomic functional annotation identified 3968 microbial KOs, among which 287 were more abundant and 961 were less abundant in CAP patients than in healthy controls (supplementary Fig. 1a). Among the most significantly altered genes, mcsA and mcsB, both of which are associated with microbial DNA repair and the stress response, presented the greatest increase in abundance in the CAP patients. This elevated abundance indicates that bacteria in the respiratory tract of CAP patients are likely experiencing substantial stress, likely due to the host immune response or antibiotic treatment. Additionally, rfbP was among the top 10 genes with significantly increased abundance in CAP patients. This gene is involved in O-antigen biosynthesis, a critical component of the bacterial outer membrane that plays a role in immune evasion, allowing pathogens to persist and cause infection. In healthy controls, genes associated with genetic mobility (insB) and nutrient metabolism (fucR, gltI, aatJ) were more abundant. This suggests a state of eubiosis in the respiratory microbiota, where a balanced microbial community is maintained without triggering pathogenic responses.

**Fig. 3:**
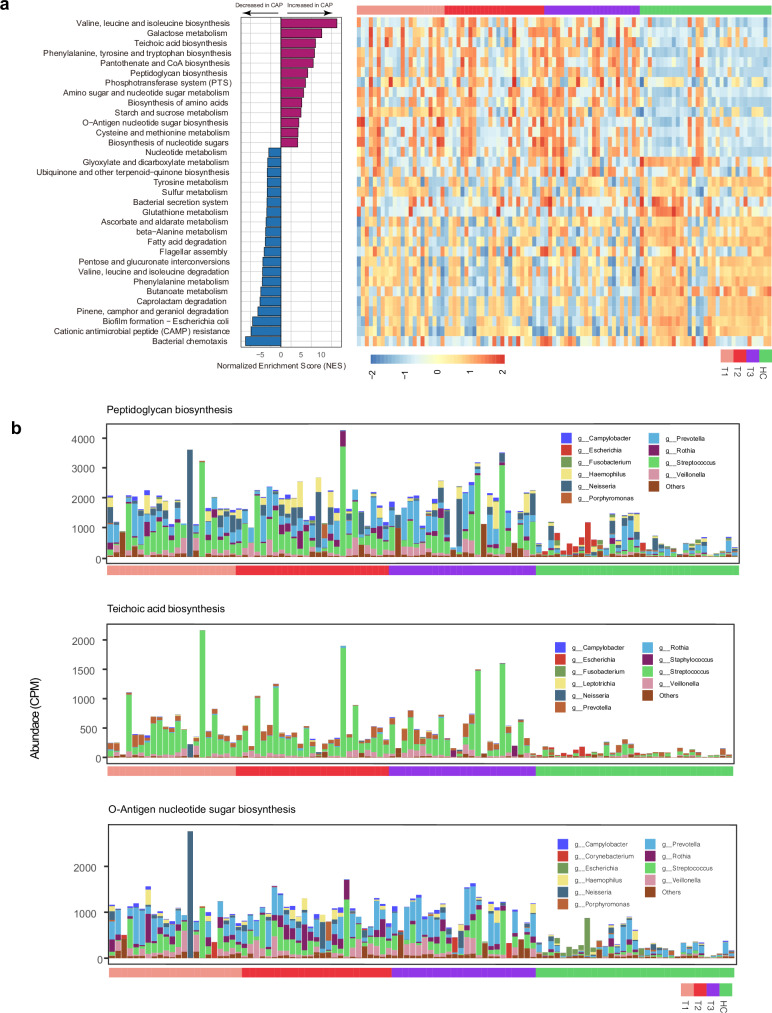
Pathogenic microbiota gene modules enriched in CAP patients. **a** KEGG pathways that are significantly enriched in CAP patients and healthy controls. The pathway enrichment score for each sample from healthy controls, three time points during the acute phase (T1, T2, T3), and the follow-up phase is shown on the right. **b** Distribution of bacterial species and their contributions to three pathways related to cell wall enhancement and host response regulation

To facilitate interpretation of CAP-associated microbial functions, we aggregated the differentially abundant KOs into KEGG pathways via KO enrichment analysis. Most of the pathways enriched in CAP patients were related to the biosynthesis of essential amino acids and energy metabolism (Fig. 3a). Notably, teichoic acid biosynthesis and peptidoglycan biosynthesis pathways were also highly enriched in the CAP-associated microbiota. These pathways are crucial for constructing and maintaining bacterial cell walls, especially in gram-positive bacteria. Additionally, pathways involved in O-antigen nucleotide sugar biosynthesis, which is the key component of the bacterial outer membrane that facilitates immune evasion, were markedly enriched in the CAP cohort. The enrichment of these pathways persisted throughout the acute phase of CAP. As HUMAnN3 employs stricter parameters and updated reference databases to improve annotation accuracy, we reanalyzed the microbial functional profiles via HUMAnN3 (v3.7) and MetaPhlAn4 (v4.0.6). Among the 33 pathways enriched through HUMAnN2 analysis, 26 were also significantly enriched according to HUMAnN3 (supplementary Fig. 3). Notably, all three Streptococcus-associated pathways enriched in CAP patients were consistently identified by both versions, supporting the reliability and reproducibility of our results.

We further explored which microbes contribute to these pathways related to bacterial cell wall reinforcement and host response interactions via functional profiles stratified by known organisms. We found that *Streptococcus* was the primary contributor to the enrichment of both the peptidoglycan biosynthesis pathway and the TEA biosynthesis pathway within the microbiota. *Streptococcus* and *Prevotella* also contributed the most to the O-antigen nucleotide sugar biosynthesis pathways. These findings indicate that *Streptococcus* plays a major role in driving enrichment at both the taxonomic and functional levels within the respiratory microbiota of CAP patients.

### Strain-level respiratory‒gut correlation of *Streptococcus* in CAP patients

**Fig. 4:**
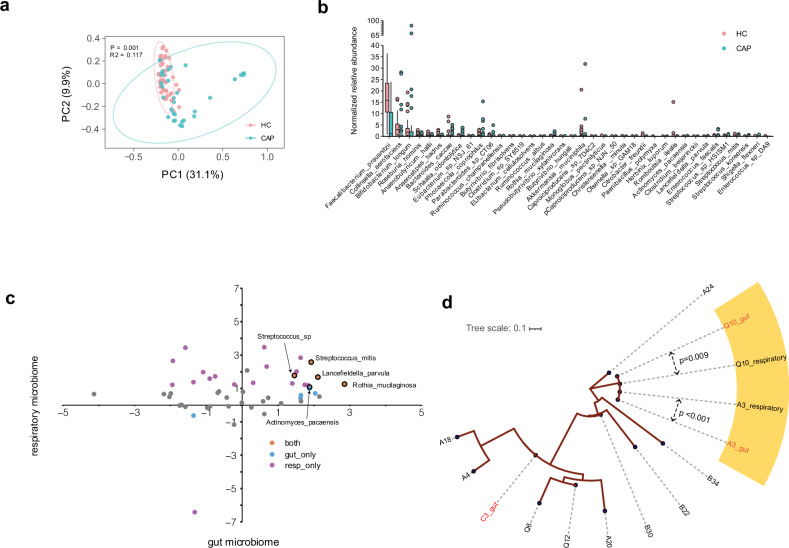
Respiratory-gut microbiota association of CAP-enriched respiratory microbes. **a** PCoA plot illustrating the distinct variation in the gut microbiota composition between CAP patients and healthy controls. **b** Box plots for the 38 differentially abundant species between CAP patients and healthy controls. The central line indicates the median, and the lower and upper hinges indicate the first and third quartiles, respectively. **c** Differentially abundant species analysis across the respiratory and gut microbiomes between CAP patients and healthy controls. Each point represents a bacterial species, classified as respiratory only (purple), gut only (blue), or both sites (orange), representing two different patterns of microbial presence: site-specific and potential transmission between sites. **d** Phylogenetic tree of *Streptococcus mitis* via strain-level analysis with StrainPhlAn. Strain identities between closely related strains are labeled. Strains with potential respiratory-gut transmission within the same patient are shaded, with gut strains labeled in red font

In addition to the respiratory microbiota, the gut microbiota was also influenced by CAP. The gut microbiota of CAP patients also showed distinct variation compared with that of healthy controls (Fig. 4a), characterized by a significant depletion of beneficial microbes such as *Faecalibacterium prausnitzii*, *Bifidobacterium longum*, and *Roseburia hominis*, which were among the four taxa with the greatest reduction in abundance in CAP patients (Fig. 4b). Notably, we observed that certain bacteria enriched in the CAP respiratory microbiota, such as *S. mitis* and *Rothia mucilaginosa*, also presented increased abundance in the gut, suggesting possible respiratory-gut transmission of these bacteria (Fig. 4c). Nevertheless, we found that no microbes depleted in the respiratory microbiota also had decreased abundance in the gut microbiota in CAP patients. We further performed strain-level profiling of *S. mitis* in both respiratory and gut microbes because of its relatively high abundance in the CAP respiratory tract. Among the 10 sputum and 3 fecal samples that had sufficient coverage for strain-level analysis, we identified highly similar strains in both niches from the same individual on day 1 (A3) and day 3 (Q10) post-hospitalization, suggesting potential respiratory-gut migration events (Fig. 4d).

### Respiratory microbiome‒host interactions in CAP patients

To explore the interactions between the respiratory microbiota and the host response in CAP patients, we performed WGCNA to identify modules of co-expressed genes and their associations with microbes. Three pairs of gene modules and microbial clusters were identified with potential associations (*p* < 0.1, correlation coefficient > 0.3), specifically, the yellow–green module with microbe cluster 3, the tan module with cluster 4, and the salmon module with cluster 5 (supplementary Fig. 1b). To identify potential bacteria‒gene interaction pathways, we performed Spearman correlation analysis to detect associations between individual genes and microbial species on the basis of host gene expression levels and microbial abundance in each significant gene‒module‒microbe‒cluster pair.

**Fig. 5:**
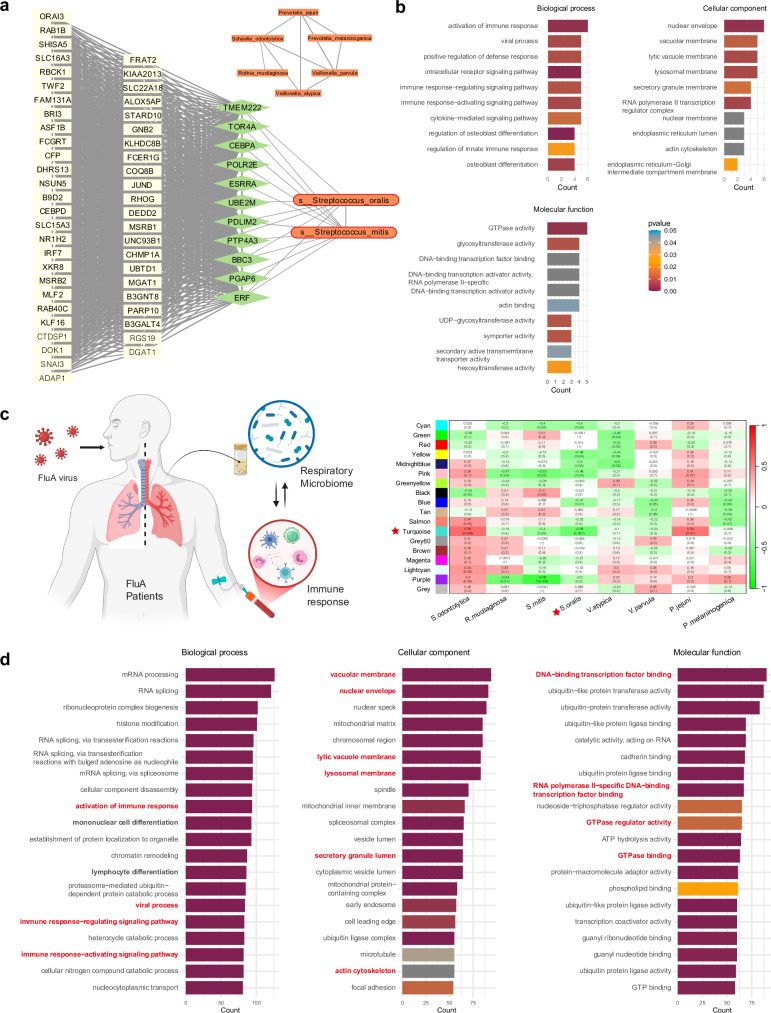
Identification of gene‒microbe interactions in CAP patients through WGCNA and Spearman correlation analyses. **a** Network visualization of the significant associations between *S. oralis* and *S. mitis* from microbial cluster 5 and genes in the salmon module. The remaining six bacteria in microbial cluster 5 formed a separate network. **b** Gene enrichment analysis of biological processes, cellular components, and molecular functions for the genes in the salmon module linked to *S. oralis* and *S. mitis*. **c** The association between bacteria in microbial cluster 5 and the host response was validated in a FluA cohort. Schematic overview and heatmap showing the correlation between bacterial species in cluster 5 and gene expression modules in the FluA cohort. Each row represents a microbial species, and each column represents a gene module identified via WGCNA. The asterisks indicate that the turquoise module was significantly associated with *S. oralis*. **d** Gene enrichment analysis of genes in the turquoise module associated with SOR in the FluA cohort. The top 20 enriched terms are shown for biological process, cellular component, and molecular function. Key immune-related pathways are highlighted in bold, and those overlapping with the original CAP cohort are additionally marked in red

The genes in the yellow–green module established no significant association with the bacteria in cluster 3, which included 6 species within the *Neisseria* genus. Microbial cluster 5 contained 8 bacteria, among which two closely related *Streptococcus* species, *S. oralis* (SOR) and *S. mitis*, established direct relationships with 6 genes in the salmon module. Gene enrichment analysis indicated that genes in this module are involved primarily in the activation of the innate immune response, virus process and cytokine response, suggesting that these two *Streptococcus* species may play a role in regulating the host immune response during viral infections (Fig. 5a, b). Similarly, microbe cluster 4, which contained 150 microbial species, exhibited a significant association between *Schaalia cardiffensis* and 5 genes in the tan module (supplementary Fig. 2a), which included 66 genes and was enriched for pathways related to lymphocyte activation and T-cell activation (supplementary Fig. 2b).

Given that more than half of this CAP cohort consisted of COVID-19 patients, we additionally enrolled 22 CAP patients with influenza A infection to validate the association between SOR and the host immune response in the context of other infections. As anticipated, we identified the turquoise model that exhibited the most significant association (lowest *p* value and highest correlation coefficient) with SOR (Fig. 5c). Subsequent gene enrichment analysis of the turquoise module revealed 6 pathways related to immune activation and regulation among the top 20 enriched pathways (Fig. 5d). Notably, the 4 pathways specifically involved in immune response activation, viral processes, and immune response-regulating/activating signaling were consistently enriched in both the original CAP cohort and the FluA cohort, highlighting the potentially universal functional role of SOR in various infection contexts.

### *S. orals* protect against influenza virus infection via rapid activation of the innate immune response

**Fig. 6:**
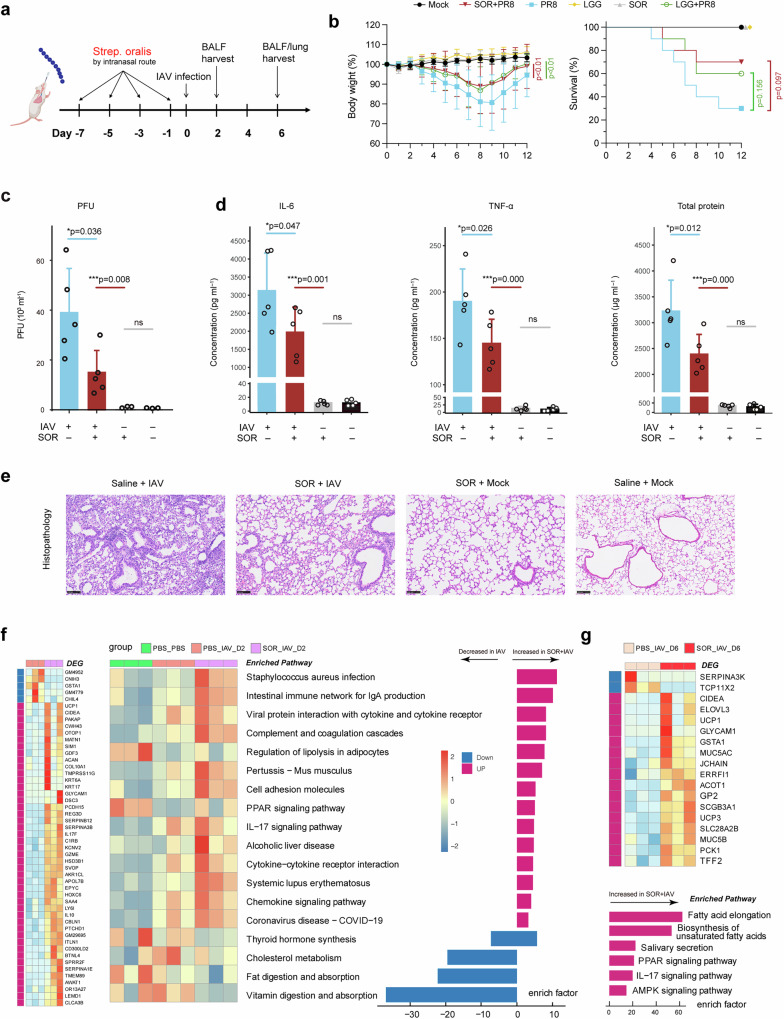
Protective effects of *S. oralis* against influenza A virus (IAV) infection in a murine model. **a** Schematic representation of the experimental design. The mice were preinoculated with *S. oralis* (SOR) or *Lactobacillus rhamnosus GG* (LGG) via the intranasal route four times prior to IAV infection. Lung and bronchoalveolar lavage fluid (BALF) samples were harvested on days 2 and 6 post-infection from a separate set of experiments. **b** Preinoculation with SOR and LGG mitigated IAV-induced weight loss and improved survival in mice. *P* values comparing SOR-infected and LGG-infected and PR8-infected mice are indicated with individual color labels (*n* = 10 mice per group). **c** The viral load in the lungs, measured as plaque-forming units (PFUs), in the four groups at 48 h post-infection. Data are shown for IAV-infected mice pretreated or not with SOR (*n* = 5 per group), and uninfected controls with or without SOR priming (*n* = 3 per group). **d** Levels of the inflammatory markers IL-6 and TNF-α and total protein concentrations in mouse BALF on day 6 post-infection (*n* = 5 mice per group). **e** The lungs of IAV-infected (IAV) and uninfected (Mock) mice with (SOR) or without (Saline) *S. oralis* pretreatment on day 6 post-infection were sectioned and stained with hematoxylin and eosin. Representative images are shown. Differentially expressed genes and pathways in the lungs of mice pretreated with *S. oralis* compared with those in the lungs of control mice on day 2 post-infection (**f**) and on day 6 post-infection (**g**). **c**, **d** Data are shown as the mean ± standard deviation (S.D.). Statistical analyses were performed via the (b survival curve) log-rank (Mantel–Cox) test, (**b** body weight curve) two-way ANOVA with Sidak’s multiple-comparison test, or (**c**, **d**) one-way ANOVA with multiple-comparison test

The microbiome–transcriptome integration analysis identified three bacteria with a positive association with the host immune response. Given that *S. cardiffensis* exhibited low abundance in both CAP patients and healthy controls (supplementary Fig. 2a), we focused our attention on the two *Streptococcus* species and their role in regulating the host immune response. Owing to the limited data on the protective role of SOR and its consistent association with immune activation pathways in both the CAP and influenza cohorts, SOR was selected for functional validation. A clinical strain of SOR was successfully isolated from one CAP patient and used to inoculate mice via the intranasal route four times prior to IAV infection (Fig. 6a). Pre-inoculation with SOR dramatically reduced IAV-induced weight loss, showing efficacy comparable to that of LGG (Fig. 6b). Notably, SOR improved the survival rates of infected mice from 30% to 70%, which was slightly greater than the 60% survival rate observed with LGG priming, although the difference was not statistically significant. Moreover, the amount of infectious viral particles in the lung was also lower at 48 h post infection than in those without SOR priming (Fig. 6c). SOR pretreatment also resulted in reduced levels of the inflammatory markers IL-6 and TNF-α in the BALF at 6 days post infection (Fig. 6d). Pathology analysis of mouse lungs harvested on day 6 post infection revealed that SOR pretreatment reduced inflammatory cell infiltration and lung jury (Fig. 6e). Additionally, SOR alleviated alveolar capillary barrier damage, as measured by the lower total protein concentration in the BALF, indicating improved permeability of the alveolocapillary membrane (Fig. 6d). To explore the mechanism underlying the protection of SOR against IAV infection, we performed transcriptome analysis of lung tissue from mice pretreated with SOR or not before IAV infection on days 2 and 6 post infection. Compared with mice without SOR treatment, pre-inoculation of SOR significantly increased the expression of numerous immune regulatory genes at day 2 post infection, including IL17F, LY6I, and C1RB, which enhanced the activation of pathways involved in bacterial infection, viral protein interaction with cytokine, cytokine and chemokine signaling, and complement response (Fig. 6f). Most of these upregulated pathways play critical roles in host defense against respiratory infections, and their heightened activation in the early stage of IAV infection inhibits virus propagation and improves outcomes in mice. However, by day 6 post-infection, when the mice began to recover, these proinflammatory genes were no longer differentially expressed between the SOR-treated and untreated groups (Fig. 6g). Similarly, the proinflammatory pathways that were highly activated on day 2 were not enriched on day 6. These findings indicate that pre-inoculation with SOR prompts timely activation of the innate immune response to effectively restrict IAV infection, and importantly, these pathways resolve rapidly in the recovery stage to prevent immune-mediated pathology.

## Discussion

CAP has long been related to the presence and activities of both cellular (bacterial, fungal) and acellular (viral) microbial pathogens with the capacity to disrupt epithelial barriers and instigate inflammatory responses. A growing body of evidence proposed that respiratory microbiota dysbiosis, especially low community diversity and enrichment of specific genera in the lower respiratory tract, is involved in pneumonia development.^8,18,19^ However, few studies have performed longitudinal metagenomic profiling of the dynamic variation in the lower respiratory microbiome from the acute phase through the recovery period. This study provides species-level and functional insights into microbiome changes from the acute phase up to four months post-discharge, as well as validation of detected host‒microbe interactions in an animal model.

Our findings, along with those of many previous studies,^6,10^ revealed that CAP patients exhibit lower alpha diversity than healthy controls do (Fig. 1b). CAP patients also demonstrated disrupted interactions between microbes, with lower edges per sample compared to health controls. Since 17 patients had received antibiotic therapy, primarily fluoroquinolones and β-lactam antibiotics, prior to the first sample collection, antibiotic usage was included as a covariate in the differential abundance analysis. We found that *Streptococcus* was the main microbe enriched in CAP patients, followed by *Veillonella*, compared with healthy controls. This finding was consistent with reviews of the lung microbiome in pneumonia patients, in which *Veillonella* and *Streptococcus* were the most abundant taxa in the sputum of adult CAP patients.^9,10^ A recent retrospective cohort study also revealed that commensal microbes, including *Streptococcus*, were the major taxa in CAP sputum microbiota and accounted for 51.2% of CAP microbial reads and 38.0% of HC microbial reads.^10^ Moreover, an increased abundance of *Streptococcus* was also observed in the gut microbiota of CAP patients, accompanied by a significant depletion of gut probiotics such as *Faecalibacterium prausnitzii* and *Bifidobacterium adolescentis*, both of which are among the three taxa with the greatest reduction in CAP patients compared with healthy controls, reflecting a dysbiotic gut microbiota, which is consistent with the findings of previous studies.^20,21^ These data suggest that the increased abundance of specific microbes in the respiratory tract may contribute to the disruption of the gut microbiota by the lung‒gut axis.

Intensive metagenomic sequencing ( > 20 G/sample) enabled detailed functional profiling of the respiratory microbiota in CAP patients. The two pathways involved in bacterial cell wall synthesis, peptidoglycan biosynthesis and teichoic acid biosynthesis, were highly enriched in CAP patients. Both peptidoglycan and teichoic acid are strong immunogens that promote the activation of monocytes/macrophages and the production of inflammatory mediators such as TNF-α, IL-6, and nitric oxide (NO).^22^ Moreover, we found that *Streptococcus* contributed predominantly to these two pathways (Fig. 3B), suggesting that members of this genus may play a role in the interaction with the host immune response. Additionally, another highly enriched pathway in CAP patients was O-antigen nucleotide sugar biosynthesis (Fig. 3B). O-antigen is a crucial component of the lipopolysaccharide (LPS) layer in gram-negative bacteria and plays a key role in microbial infections.^23^ Our findings indicate that *Streptococcus* also contributes to this pathway, possibly through the pneumococcal group O antigen (C-polysaccharide) produced by some *Streptococcus* species, such as *S. mitis* and SOR.^24,25^ These findings indicate that respiratory commensal microbes such as *Streptococcus* contribute significantly to the microbial pathways that potentially activate the host immune response in CAP patients.

Integration of the respiratory microbiome and the host response revealed two gene modules related to respiratory microbes. As anticipated, the first gene‒bacteria link was established by SOR and *S. mitis*, which are connected to genes involved in innate immune response activation and viral processes (Fig. 5a). This finding aligns with their high abundance, enrichment in pathogen-associated microbial pathways, and ability to be transmitted from the respiratory tract to the gut in these patients (Fig. 4). The role of *Streptococcus* species in driving host immune responses, particularly through the activation of innate immunity, is considered a mechanism by which upper respiratory tract (URT) commensal bacteria regulate pathogen acquisition and invasion.^26^ However, its interaction with the host response in the lower respiratory tract has rarely been described.

Given its high abundance and association with immune activation pathways in both the CAP and FluA cohorts, SOR was isolated from a CAP patient, and its interaction with the host response was validated in an animal model (Fig. 6a). As a comparison, we incorporated LGG in mice experiment, a well-studied probiotic that has shown protective efficacy against both respiratory viral and pneumococcal infections in previous studies.^27–29^ We used 1 × 10⁵ CFU for intranasal intervention, as previous studies have indicated that this dosage of bacterial intervention has a biological effect.^30,31^ SOR demonstrated comparable efficacy to LGG in mitigating body weight loss and exhibited superior performance in improving survival rates (Fig. 6b). Additionally, SOR pretreatment also reduced lung inflammation, alleviated lung injury, decreased the viral load, and lowered the levels of inflammatory markers such as IL-6 and TNF-α (Fig. 6c, d, e).

Transcriptome analysis revealed that SOR priming induced significantly increased levels of the innate immune response, which is typically activated during respiratory infections in the early stage of IAV challenge. Specifically, SOR pre-inoculation upregulated the expression of the IL17F gene and robustly activated pathways related to bacterial infection, lgA production, virus protein interaction with cytokine, complement response, and IL-17 signaling pathways during influenza virus infection. Similarly, another study also showed that a single aspiration of three oral commensals, including *S. mitis*, induced a prolonged Th17 response that mitigated susceptibility to *S. pneumoniae*.^16^

Notably, IL-10 was also upregulated by SOR treatment, which may play a role in lowering the excessive immune response to prevent immune-mediated lung damage. The dual role of respiratory commensals in activating innate immune responses while concurrently regulating lung inflammation through IL-10 was also observed with *Prevotella*, which promotes neutrophil activation and mitigates the harmful effects of inflammation through IL-10 regulation when coinfected with *S. pneumoniae* in mice.^15^ Moreover, the highly activated proinflammatory genes and pathways decreased to levels comparable to those in mice without SOR pre-intervention, with no significant difference between the two groups by day 6 post‒infection, when the mice began to recover weight. These dynamic variations in the host response in mice indicate that pre-inoculation with SOR can induce timely activation of the innate immune response to combat infection and, more importantly, rapid resolution of inflammation as the infection is brought under control when recovery begins, thereby preventing immune-mediated damage from excessive inflammation.

This study has several limitations. First, the sample size in this study was relatively small for the challenge of collecting longitudinal samples from hospitalized CAP patients and following them up for several months; the results for follow-up patients may need larger cohort validation. Second, owing to difficulties in recruiting healthy individuals during the peak respiratory virus season, we used healthy controls of comparable age from a previous study, which may have introduced batch effects. This limitation was partially mitigated by the observation of similar enriched taxa in an independent study,^10^ cross-validation of the microbe–host associations in a newly recruited influenza (FluA) cohort, and further validation in an animal model. Third, although induced sputum is widely used as a surrogate to study the lung microbiome, it may be contaminated by microbes from the upper respiratory tract, particularly when explaining the significance of microbes with low abundance in the sputum, such as *S. cardiffensis*, detected in this study.

Our results demonstrated that dysbiosis in the lower respiratory tract microbiota persisted throughout the acute phase and possibly did not fully recover even four months post‒discharge. Pre‒inoculation of SOR in mice induced a potent innate response against respiratory virus infections and resolved timely to prevent immune-mediated damage, demonstrating protective efficacy comparable to that of the well-established probiotic LGG. Our findings highlight the airway microbiota as an important immune regulatory component that contributes to respiratory tract health.

## Materials and methods

### Study design

Patients were diagnosed with CAP based on the clinical practice guidelines of the Chinese Thoracic Society.^32^ Accordingly, CAP was diagnosed if disease onset occurred in the community and chest radiograph revealed new patchy infiltrates, lobar or segmental consolidation, ground-glass opacities, or interstitial changes, with or without pleural effusion. In addition, patients presented at least one of the following clinical manifestations: (1) new onset of cough or expectoration or aggravation of existing symptoms of respiratory tract diseases, with or without purulent sputum, chest pain, dyspnea, or hemoptysis; (2) fever; (3) signs of pulmonary consolidation and/or moist rales; and (4) a peripheral white blood cell count (WBC) > 10 × 10^9^/L or < 4 × 10^9^/L, with or without a left shift. Patients with chronic respiratory diseases such as chronic obstructive pulmonary disease (COPD), bronchiectasis, or asthma were excluded. This study did not involve any clinical intervention or investigation of clinical outcomes and thus was not registered as a clinical trial.

Only hospitalized CAP patients aged 18 years and above were enrolled in this study from January to April 2023, during a period with a high prevalence of respiratory infections. A series of clinical samples, including sputum, whole blood and stool, were collected within 24 h (T1) after admission, followed by a second collection at 2–3-day intervals (T2) and a final collection 1–2 days before discharge (T3) (Fig. 1a). Patients were followed up for 4 months after discharge, and samples were collected if they reported no respiratory symptoms and no use of antibiotics within 1 month. Sputum specimens with < 10 squamous epithelial cells and >25 polymorphonuclear neutrophils per low power (microscopic) were considered suitable. Owing to the risk of exposure to circulating respiratory viruses when healthy individuals are recruited to the hospital for induced sputum collection, we used sputum samples from a previous study as controls, which included 33 healthy individuals with ages comparable to those in our cohort.^30^ All these health controls reported no respiratory symptoms and no antibiotic use within the past month.^30^ The sputum samples were liquefied with 0.1% dithiothreitol solution, and aliquots were stored at -80 °C until they were transported to the laboratory for processing within 3 months. The stool was passed directly into a clean container placed under the seat of the toilet and stored at -80 °C immediately. Stool samples were collected from 50 healthy volunteers of local community origin when they visited for routine health checkups to serve as gut microbiota controls; these individuals had no history of gastrointestinal disorders and had not used antibiotics for at least one month. Whole blood was collected into PAXgene Blood RNA tubes (BD, New York, USA) and then transferred to −20 °C for storage. This study was approved by the ethics committees of Zibo Municipal Hospital and Japan-Friendship Hospital.

### Influenza patient cohort (FluA cohort)

Given that the above patients were enrolled during a period of COVID-19 incidence, we additionally enrolled a cohort of patients with CAP due to influenza A infection between February and April 2025. Influenza infection was confirmed via real-time PCR testing of influenza A and B viral RNA via swabs collected within 24 h of hospitalization. Sputum and blood samples were obtained via the methods described above.

### Clinical microbiology test

Expectorated sputum was used for bacterial, mycobacterial, and fungal cultures. *M. tuberculosis* and rifampicin resistance were detected via the rapid molecular test Xpert MTB/RIF (Xpert). Oropharyngeal swabs were collected from all patients within 24 h of hospitalization, followed by SARS-CoV-2 nucleic acid testing via real-time PCR. To cover a broader spectrum of potential pathogens, at least one sputum sample from each patient, collected within 24 h of hospitalization, was analyzed via a targeted NGS test encompassing 198 respiratory pathogens, including viruses, bacteria, mycoplasma/Chlamydia, and fungi (supplementary Table 1).

### Metagenomic sample processing and sequencing

The sputum samples used for metagenomic sequencing were collected separately from those used for routine clinical microbiology. DNA was extracted via DNeasy PowerSoil Pro Kits (QIAGEN, German), which include mechanical lysis via beads to ensure lysis of all types of microbes. The gut microbe DNA was extracted with the same kit with no preprocessing using 250 mg of stool immediately after thawing from frozen storage. DNA sequencing libraries were prepared via the QIAseq FX DNA Library Kit (QIAGEN, German) and sequenced via the Illumina HiSeq 4000 platform with paired-end reads of 150 bp in length. Four reagent controls (DNA extraction blanks) were included for sequencing.

### Metagenomic analysis

#### Taxonomic profiling of metagenome data

Metagenomic reads were subjected to quality control via fastp v0.21.0^33^ to remove low-quality reads, which removed reads with more than 40% of bases having a Phred score below 20 and reads shorter than 100 bp after trimming. In addition, reads with excessive ambiguous bases (N) or adapter contamination were also filtered out. Host-derived reads were subsequently removed via kneaddata v0.12.0.^34^ The resulting reads, referred to as the ‘clean data’, were deposited in the Genome Sequence Archive (GSA) under the accession ID CRA019395. These clean data were used for subsequent analyses in this study. Metagenomic library coverage was estimated for each sample via Nonpareil 3 with the option “-T kmer” on all the clean reads.^35^ Species-level profiling was performed via Kraken2 v2.1.3 with the PlusPF database containing archaeal, bacterial, viral, plasmid, human, protozoan and fungal data released in December 2022.^36^ Only species with relative abundances greater than 0.01% in at least 20% of the total samples were retained. The bacterial species detected in at least two of the four reagent controls with a relative abundance greater than 0.001 were considered potential contaminants and were excluded from further analysis. None of the species identified as reagent contaminants significantly differed between the CAP and control groups, nor were they associated with the host response.

### Microbiome analysis

To evaluate microbiome alterations associated with CAP, sputum microbiome features were compared between CAP patients and healthy controls to identify microbial taxa and network features that are disrupted during infection. Microbe alpha diversity was calculated via the vegan v2.6-4 package in R v4.3.0. Tests for differences in beta diversity were performed via PERMANOVA, as implemented in R’s vegan package, using Bray–Curtis distances at 1000 permutations. The association of the microbiome with CAP was performed via a general linear mixed model (GLMM) in the R lme4 package, adjusting for age and antibiotic usage as covariates, with subject identity included as a random effect. Spearman correlation analysis was performed between all pairs of bacterial species, and significant correlations (*P* < 0.05) were retained for co-occurrence network construction via the R igraph v1.4.2 package. The number of significant correlations in each group was normalized by the number of samples in that group to facilitate the comparison of edge density across groups. A cutoff of 0.5 for absolute Spearman’s rho was implemented for network visualization in Gephi software.^37^

### Microbiome functional analysis

The functional profiles of respiratory microbes were estimated via Humann2,^38^ which uses marker species information from MetaPhlAn2 to align reads to species pangenomes. Gene families were then identified via the UniRef90 database, regrouped into KEGG Orthogroups (KOs) and normalized according to relative abundance. Differential KOs between groups were identified via the limma v3.56.1 package, and those with FDR-adjusted *p* values < 0.1 and a fold change >2 were retained. Microbial pathway enrichment was then performed via the enrichKO function in the MicrobiomeProfiler v1.11.0 package.^39^ The pathway enrichment score for each sample was also calculated via the GSEA function in the GSEABase v1.62.0 package.^40^ As HUMAnN3 was universally used and was considered stable at the time of our analysis, we also reperformed the functional analysis via HUMAnN3 (version 3.7) to confirm the robustness of the functional analysis results.

### Bacterial strain-level analysis and potential oral-to-gut transmission

The potential oral-gut microbiome crosstalk in CAP patients was investigated through strain-level comparisons of sputum and stool microbiota. Only patients with paired sputum and stool samples were included in this analysis. Strain-level profiling was performed with StrainPhlAn 4 via the custom species-level genome bin (SGB) marker database, with the parameters ‘--marker_in_n_samples 1 --sample_with_n_ markers 5 --phylophlan_mode accurate --mutation_rates’. The phylogenetic distance between each strain was calculated via PyPhlAn. Oral-to-gut shared strains were defined as pairs of respiratory and gut samples from the same individual collected on the same day with phylogenetic distances less than 0.03, as recommended by StrainPhlAn.

### Integrated analysis of the host response and respiratory microbiome

#### Whole-blood transcriptomics analysis

Whole blood samples were collected and stored in PAXgene Blood RNA Tubes (QIAGEN). Total RNA was extracted via the PAXgene Blood RNA Kit (QIAGEN), followed by mRNA sequencing on the Illumina NovaSeq 6000 platform with paired-end reads of 150 bp. The raw reads were quality filtered via Fastp v0.20.0 and aligned to the human genome GRCh38 via HISAT2 v2.1.0. The gene count matrix was then summarized via the FeatureCounts v4.8.1 package.

### Microbiome feature extraction

The respiratory microbiota at the species level was grouped into several clusters based on their abundance via the K-means algorithm from the R Cluster package v2.1.4. To determine the optimal number of clusters, we employed two methods: the elbow method using the total within-cluster sum of squares and the gap statistic. The elbow method helps identify the point where the total within-cluster sum of squares starts to level off, suggesting the ideal number of clusters. The gap statistic provides a comparative measure of the clustering gaps for different values of k, with the optimal number identified at k = 7. For the microbes in each cluster, we performed principal component analysis (PCA) on the species abundance to obtain the first principal component (PC1) value for each cluster. PC1 captured the dominant variation within each cluster and was used in subsequent association analyses to reduce the dimensionality of the microbiome data.

### Integrative transcriptome‒microbiome analysis

The gene abundance matrix from host transcriptomics was subjected to co-abundance clustering via WGCNA v1.72.^41^ We selected the top 6000 most highly expressed genes to generate gene modules. This involved constructing a sample clustering tree, identifying outliers, and determining the soft-thresholding power for network construction. The optimal power for achieving scale-free topology was selected, and a gene co-expression network was built, resulting in multiple gene modules. To explore the potential associations between microbes and gene modules, the PC1s of the seven microbiome clusters were input into the WGCNA as traits. Then, Pearson correlation analysis was performed on each module and microbiome trait, allowing the identification of gene modules that were significantly associated with microbiome clusters. Genes clustered in the module of interest were subjected to KEGG pathway enrichment analysis via clusterProfiler v4.8.1.^39^ For gene‒module and microbiome‒cluster pairs with significant associations, Spearman correlation analysis was performed between the genes in that module and the species in that cluster on the basis of their abundance data, followed by occurrence network construction and visualization as described above.

To investigate the interaction between the host response and specific bacteria, the relative abundance of that bacteria was extracted and used as a trait in the WGCNA as described above. Gene modules with significant associations were further analyzed for gene pathway enrichment to identify the pathways regulated by that bacterium.

### In vivo murine model assays

#### Virus

Influenza virus strain A/Puerto Rico/8/1934 (H1N1) was inoculated into the chorioallantoic fluid of 9-day-old specific-pathogen-free (SPF) chicken embryos. The titer of a virus stock was calculated in plaque-forming units (PFUs) via plaque assays, and the median lethal dose (LD50) was determined in adult mice via intranasal inoculation. All viral infection experiments were performed in a biosafety level 2 laboratory following standard operating protocols.

### Bacterial species isolation and inoculation

Integrative transcriptome–microbiome analysis revealed that both *Streptococcus oralis* (SOR) and *Streptococcus mitis* are associated with host immune activation pathways (detailed in the Results section). *S. mitis* has been studied in several published studies that induced Th17 responses to protect against *S. pneumoniae* infection,^16,42^ but the role of SOR in infection remains largely unexplored. Given this gap and the consistent association of SOR with immune activation pathways in both the original and the new FluA cohorts (detailed in the Results section), we selected SOR for functional validation in murine models. SOR was isolated from the sputum of a CAP patient and cultured via brain heart infusion. *Lactobacillus rhamnosus GG* (LGG, ATCC53103), a respiratory probiotic known for its protective efficacy against influenza virus infection when administered intranasally prior to challenge in neonatal mice,^27^ served as a positive control. Then, 1×10^5^ colony-forming units of bacterial cells were resuspended in 30 μl of saline and administered intranasally to the mice every 2 days for a total of four doses prior to the establishment of the pneumonia model. Saline was used as a control and was administered intranasally at the same frequency. A total of 10 mice were used per group.

### Viral infection of mice

All the mice used in this study were 8–9-week-old female C57BL/6 mice. The mice were anesthetized before intranasal inoculation with 10^3^ PFU of A/Puerto Rico/8/34 (PR8) in 30 μl of PBS (1 LD50). The mice were weighed and monitored every two days and euthanized for bronchoalveolar lavage fluid (BALF) and lung sampling on Days 2 and 6 post-infection. The infected mice with more than 20% weight loss were considered dead. All mouse experiments were approved by the Ethics Committee of China-Japan Friendship Hospital and Beijing Laboratory Animal Research Center (2023-KY-066).

### Lung histopathology

Lungs were collected from the mice on day 6 post-infection to evaluate their inflammation status. Upon sampling, the mouse lungs were immediately fixed with 10% buffered formalin for at least one week at 4 °C, processed, embedded in paraffin, sectioned, and then stained with hematoxylin and eosin (H&E).

### Inflammation biomarker measurement and virus titration

BALF was collected from the mice by injecting 1 ml of cold PBS into the trachea via a 20-G needle. After three instillations and withdrawals, the BALF supernatant was collected by centrifugation at 1500 × *g* for 10 min at 4 °C. The levels of IL-6 and TNF-α were measured via enzyme-linked immunosorbent assay (ELISA) kits (Thermo Fisher) according to the manufacturer’s instructions. The total protein concentration of the BAL fluid, which reflects an increase in the permeability of the alveolocapillary membrane, was measured via the Rapid Gold BCA Protein Assay Kit (Thermo Fisher). For virus titering, the lung was homogenized in 1 ml of PBS and centrifuged at 400 × *g* for 5 min at 4 °C. The supernatants containing influenza virus were titered via plaque assays.

### Lung transcriptomic analysis

The lungs were weighed and homogenized, and RNA was extracted via a RNeasy Mini Kit (QIAGEN) according to the manufacturer’s protocol. The extracted RNA was then subjected to mRNA sequencing, and the gene expression matrix was obtained via the pipeline described above. Differentially expressed genes were identified via edgeR with an FDR value < 0.1 and a fold change >2, followed by gene enrichment analysis via the clusterProfiler v4.8.1 package. The pathway enrichment score was estimated for each sample via the GSVA v1.48.0 package and visualized via the ggplot2 v3.4.2 package.

### Statistical analysis

Multivariate analyses associated with the respiratory microbiome and CAP for longitudinally collected samples were performed via a general linear mixed model (GLMM) in the R lme4 v1.1.33 package. Age and antibiotic usage was included as a covariate, and subject identity was treated as a random effect. *P* values were corrected via the false discovery rate (FDR) method, and species with an FDR < 0.1 and a fold change >2 were considered significantly associated. A statistically significant difference was determined by two-tailed Student’s *t*-test for normally distributed data or the Mann‒Whitney test for non-normally distributed data. For comparisons involving three or more groups, one-way ANOVA was applied, followed by post hoc analysis via Tukey’s test for all pairwise comparisons or Dunnett’s test when multiple groups were compared against the control group.

## Supplementary information

supplementary data

pathogen spectrum of tNGS and major_tax_percentage

sample and patient info

contamination species excluded from microbiome analysis

## Data Availability

The authors declare that the data supporting the findings of this study are available within the article, its supplementary information files or Source Data file. The human-removed metagenomic sequencing data generated in this study have been deposited in the Genome Sequence Archive (GSA) under the accession ID CRA019395. The human whole blood transcriptomics and mice lung transcriptomic reads were deposited in GSA under the accession ID HRA012220 and CRA027578, respectively.

## Code availability

The primary code for generating the results of the study is available on the GitHub repository (https://github.com/Xiaohui-Z/CAP_Respiratory-microbiome).
