# Supplementary material for: Enrichment of Streptococcus oralis in respiratory microbiome enhance innate immunity and protects against influenza infection
Source: Signal Transduct Target Ther. 2025 Aug 27;10:272. doi: 10.1038/s41392-025-02365-x (PMC12381301; doi:10.1038/s41392-025-02365-x)
Supplement: Supplementary file 1 — supplementary data [file 41392_2025_2365_MOESM1_ESM.pdf]

Supplementary Materials for

Enrichment of *Streptococcus oralis* in respiratory microbiome enhance innate immunity and protects against influenza infection

Xiaohui Zou, Hongyun Cao, Lizhe Hong, Lijun Suo, Chun Wang, Kang Chang, Yawen Ni, Bo Liu, Bin Cao

Correspondence to Bo Liu (lwmx@foxmail.com) and Bin Cao (caobin_ben@163.com)

This PDF file includes:

Figure S1 to S3

Other Supplementary Materials for this manuscript include the following:

Table S1 (Excel document)

Table S2 (Excel document)

Table S3 (Excel document)

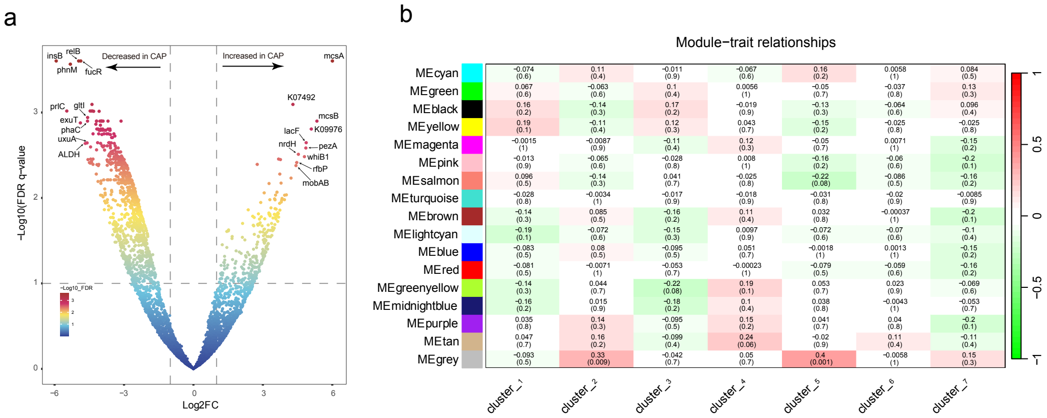

Figure S1. Respiratory functional and host–microbe interaction analysis.

(a) Volcano plot displaying differentially expressed KEGG orthogroups (KOs) between CAP patients and healthy controls. The x-axis represents the log2-fold change (log2FC), and the y-axis represents the -log10 of the FDR-adjusted q values. The top 10 KOs with the largest fold changes in CAP patients and healthy controls are labeled. (b) Correlation heatmap between host gene modules and microbiome clusters. The color scale represents the strength of the Pearson correlation coefficient, with red indicating positive correlations and green indicating negative correlations. The numbers in each cell represent the correlation coefficient, with corresponding p values in parentheses.

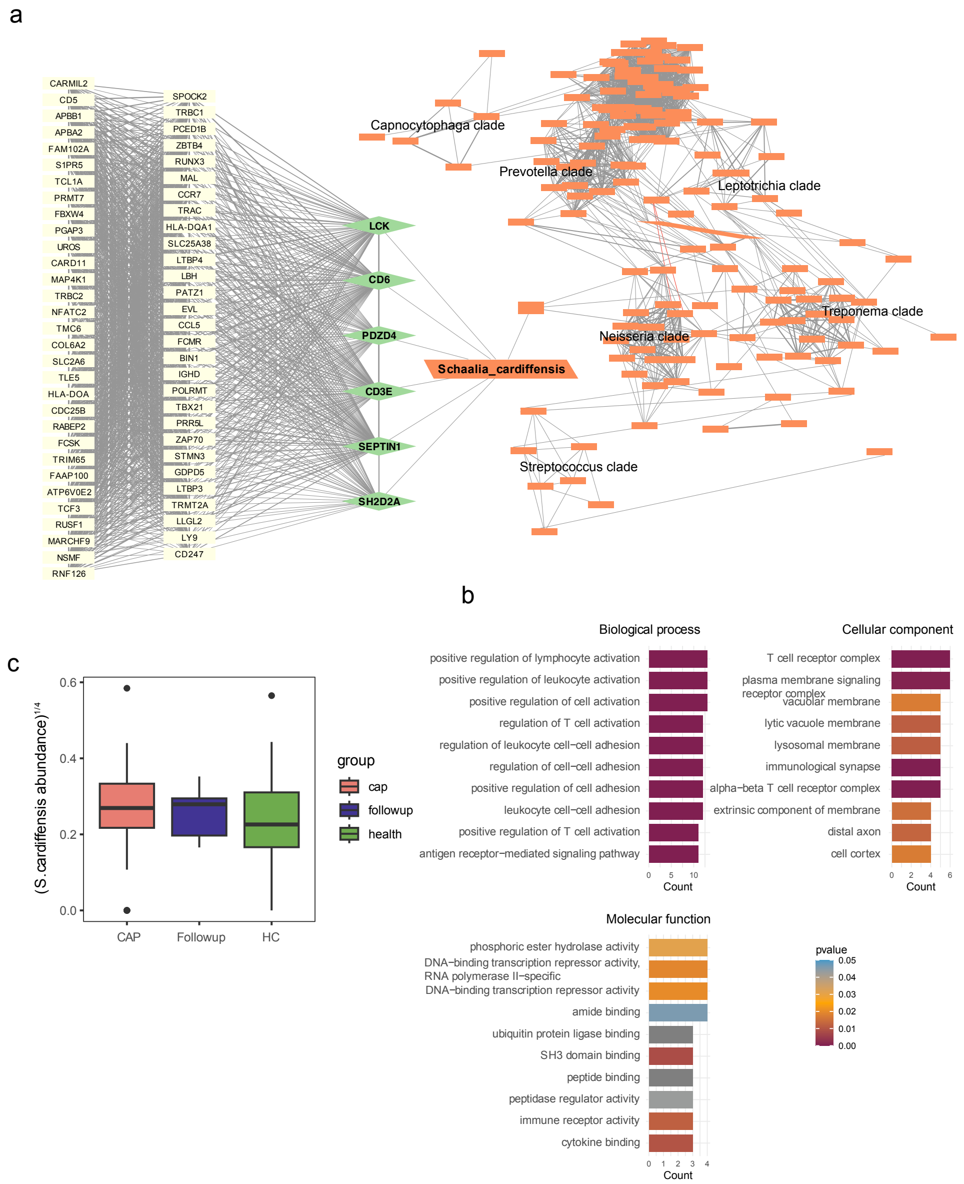

Figure S2. Associations between *Schaalia cardiffensis* and host gene modules in CAP patients.

(a) Network visualization of associations between taxa from microbial cluster 4 and genes in the tan module. *S. cardiffensis* was the only microbe that established a significant association with genes in the tan module. The dominant genera in each microbial subclade are annotated, including *Capnocytophaga, Prevotella, Neisseria, Treponema, Leptotrichia*, and *Streptococcus*. Each node represents a gene (left) or microbe (right), and edges indicate significant correlations. (b) Gene ontology (GO) enrichment analysis of biological processes, cellular components, and molecular functions for genes in the tan module associated with *S. cardiffensis*. (c) The relative

abundance (fourth root-transformed) of *S. cardificensis* across CAP patients, follow-up patients, and healthy controls. No significant differences were observed among the three groups.

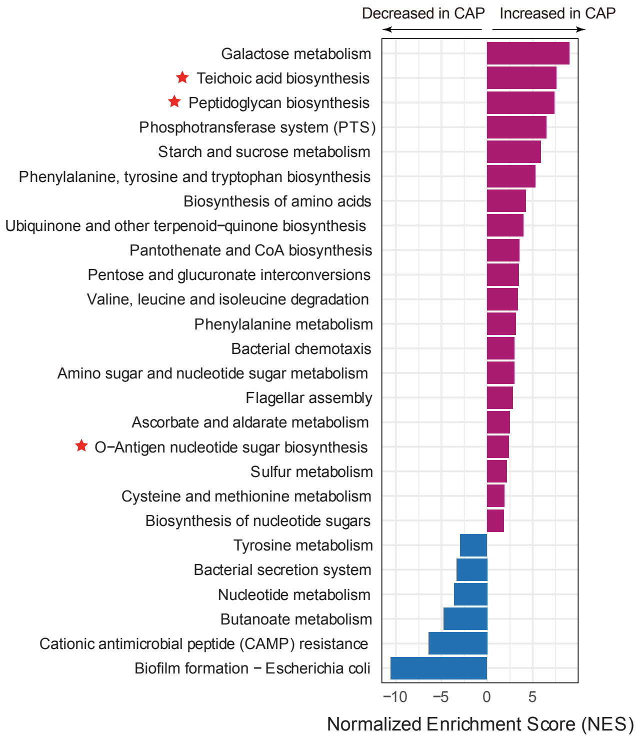

Figure S3. Microbial functional profiling via HUMAnN3 (v3.7) and MetaPhlAn4 (v4.0.6) in CAP patients and healthy controls.

Most microbial pathways enriched in HUMAnN2 analysis were also significantly enriched when HUMAnN3 was used (26/33). Key pathways related to bacterial cell wall biosynthesis and immune evasion—teichoic acid biosynthesis, peptidoglycan biosynthesis, and O-antigen nucleotide sugar biosynthesis—are marked with red stars.
